# Racial Disparities in Parkinson Disease Clinical Phenotype, Management, and Genetics: Protocol for a Prospective Observational Study

**DOI:** 10.2196/60587

**Published:** 2025-04-07

**Authors:** Deborah A Hall, Josh M Shulman, Andrew Singleton, Sara Bandres Ciga, Michelle Hyczy S Tosin, Bichun Ouyang, Lisa Shulman

**Affiliations:** 1 Department of Neurological Sciences Rush University Chicago, IL United States; 2 Departments of Neurology and Molecular and Human Genetics Baylor College of Medicine Houston, TX United States; 3 Laboratory of Neurogenetics, National Institutes of Aging National Institutes of Health Bethesda, MD United States; 4 Center for Alzheimer’s and Related Dementias National Institute on Aging and National Institute of Neurological Disorders and Stroke National Institutes of Health Bethseda, MD United States; 5 Department of Neurological Sciences Rush University Medical Center Chicago, IL United States; 6 Department of Neurology University of Maryland School of Medicine University of Maryland Baltimore, MD United States

**Keywords:** Parkinson disease, racial disparities, clinical protocol, health disparities, genetic risk factors, quality of life, quality of care

## Abstract

**Background:**

Parkinson disease (PD) has been described and studied extensively in White populations, with little known about how the disease manifests and progresses in patients from the Black community. Studies investigating disease features in Black populations are uncommon, with some suggesting that the Black population with PD is more disabled and has greater disease severity and different clinical features compared with the White population with PD. These health disparities are likely to influence the quality of care for Black patients with PD.

**Objective:**

This study aimed to investigate the motor and nonmotor symptoms and quality of life in Black and White participants with PD in a case-case design.

**Methods:**

This is an observational, prospective, multicenter, case-case design study. Other aims will investigate the management of PD in Black individuals and the presence of shared or unique genetic risk factors among the Black PD population. A total of 400 Black and 200 White participants with PD will be recruited. Data will be collected at 7 US sites and entered into a Research Electronic Data Capture database. Linear multivariate regression analysis will be used, except for comparing PD management, which will be analyzed using the chi-square test or Fisher exact test. Bonferroni correction will be applied. This protocol also describes plans for educational programming for clinicians and patients at the end of the study in partnership with national PD organizations.

**Results:**

The Rush Institutional Review Board approved the project as the single-site institutional review board in February 2022, and it was funded by the National Institute of Neurological Disorders and Stroke in April 2022. Recruitment began in July 2022. At the time of submission of this manuscript, 131 participants had been recruited.

**Conclusions:**

To our knowledge, this is the largest study of PD phenotype and management in Black patients in the United States. The planned collaboration with the Global Parkinson’s Genetics Program and PD GENEration will enhance our understanding of genetic risk factors for PD in this understudied population.

**International Registered Report Identifier (IRRID):**

DERR1-10.2196/60587

## Introduction

### Background

Parkinson disease (PD) is a progressive and incurable neurodegenerative disorder affecting 1 million people in the United States [1]. Health care resource usage costs for patients with PD in the United States are high and rise 2 to 3-fold in individuals with advanced disease [2]. Our current understanding of PD is disproportionately based on studying populations of European ancestry, leading to a significant gap in our knowledge about the clinical characteristics, life experiences, functional outcomes, and pathophysiology in individuals of African descent. The cumulative incidence of PD in African Americans has been estimated at 23/100,000, compared with 54/100,000 in European Americans [3]. Direct comparison of Black and White patients suggests greater disability and disease severity in Black individuals [4], and our data suggest that quantitative measures may be more sensitive in detecting these differences [5]. Factors proposed to account for phenotypic differences include barriers to access to care and methodological confounds due to inconsistent diagnostic criteria or ascertainment bias [6-8]. It is also possible that population-specific genetic variation modifies PD risk and clinical manifestations of PD in Black individuals. Approximately 90 common susceptibility loci for PD and a growing number of rare gene variants are now well-established in White populations [9]. Still, the impact of these factors on the Black population is largely unknown.

In our pilot work, Black and White patients with PD participated in a clinical phenotyping study, and the results showed no significant differences between the groups in sex, education, disease duration, Hoehn and Yahr stage, or Movement Disorder Society-Unified PD Rating Scale (MDS-UPDRS) part III Motor scores [10]. However, quantitative NIH Toolbox performance measures detected differences in gait and balance between Black and White participants with PD: gait speed (0.8±0.3 vs 1.1±0.2, *P*<.001), pegboard (41.4±15.6 vs 33.2±10.9, *P*=.04, and standing balance (32.7±13.1 vs 47±12, *P*<.01) [5]. In the nonmotor assessments, the Montreal Cognitive Assessment (23.4±3.1 vs 27±2.1, *P*<.005) and Symbol Digits Modalities Test (39.4±14.1 vs 49.6±9.1, *P*=.01) scores were worse in Black participants after correcting for education level. Hamilton Depression scale scores were worse in Black participants with PD compared with White participants (7±5.6 vs 4±3.5, *P*=.04), but other neuropsychiatric scales were similar between groups. The PD Quality of Life (PDQ-39) scores were higher (worse) in Black participants with worse ratings in participation in social roles and activities.

In summary, this pilot data showed differences in the motor examination, nonmotor features, and quality of life of Black patients with PD that warranted the current larger study. In our previous work and this protocol, race is self-identified by the patient as Black or White. Traditionally in the Chicago metropolitan area, this includes Black patients who are African, Afro-Caribbean, African American, or who report a mixture of races.

Racial disparities in the clinical management of PD include inequitable access to care and disparities in therapeutic interventions. Previous studies show that Black individuals with parkinsonism are less likely to see a neurologist, have less access to telemedicine, and are less likely to receive treatment, including antiparkinsonian medication, surgical procedures, and rehabilitation therapy. Black patients are 30% less likely to see an outpatient neurologist for neurologic conditions [11], and both Black persons and those with lower socioeconomic status are less likely to receive specialized care for PD [4]. Compared with White persons with PD, Black patients with PD were 40% less likely to receive any rehabilitation therapy (physical therapy, occupational therapy, and speech therapy). Black persons with parkinsonism were less likely to be receiving any antiparkinsonian medication on their initial visit to a movement disorders center [12]. They were half as likely to receive newer antiparkinsonian medications but twice as likely to be on antipsychotic medication. African Americans were 4 times less likely to receive any treatment for PD (medication or physical therapy) in a cohort with the same health care insurance (Medicaid) [13], and White persons were nearly twice as likely to be prescribed medications for PD in a study of racial disparities in stroke (National Institute of Neurological Disorders and Stroke [NINDS] Reasons for Geographic and Racial Differences in Stroke study) [14]. African Americans were also found to be 5 to 8 times less likely to undergo deep brain stimulation surgery for PD than White patients [15,16]. Notably, racial disparities in access to care and clinical management are likely to be associated with adverse outcomes, including greater severity of Parkinsonian symptoms and greater disability [4].

Genetic susceptibility loci and genetic variants causing monogenic PD have been explored in populations of European, Latino, and Asian ancestry [17]. Based on preliminary work, it appears that the cumulative genetic risk for Black and African American patients with PD shows significantly different distributions compared with European populations when applying the genetic risk score composed of the 90 risk loci previously linked to European populations [17].

More recent work from the Global Parkinson’s Genetics Program confirms this differential risk, identifying a novel genetic risk factor in *GBA1* in patients with PD of African ancestry [18]. This supports the need for additional work in this area.

### Objective

The overall objectives of this study are to perform comprehensive phenotyping, compare Black and White persons with PD, and investigate causes of racial disparities, including differences in clinical management and responsible genetic risk factors. The study is partnering with the ongoing Global PD Genetics Program (GP2) [19], which contributes samples and granular phenotypic data from the enrolled participants to enhance our understanding of the genetic architecture of PD in the Black population. The long-term goal of this application is to address the critical gap in knowledge of PD in this underserved population to improve diagnosis, optimize treatment, and plan for clinical trials. The central hypothesis is: PD is phenotypically and genetically different in Black versus White populations, and Black patients receive different and suboptimal clinical management. The research questions this study hopes to answer are as follows: (1) whether Black participants with PD have worse quantitative motor function and cognition, higher levels of depression and disability, and reduced quality of life compared with White participants with PD; (2) whether pharmacological, surgical, rehabilitation, mental health, and telehealth interventions differ by race with underuse of newer or more costly interventions in Black participants with PD; and (3) whether the contribution of genetic factors to PD risk and heterogeneity differs by race.

## Methods

### Overview

**Figure 1:**
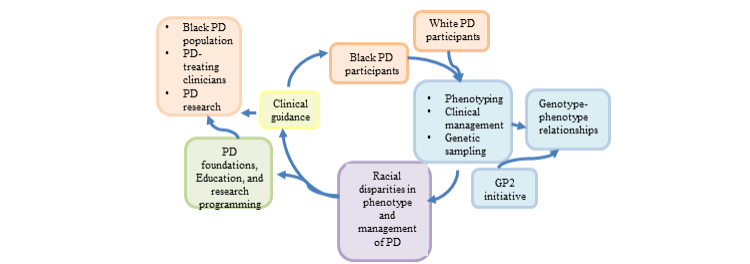
Conceptual model. GP2: Global Parkinson’s Genetic Program; PD: Parkinson disease.

The overall strategy is to describe clinical phenotypes, genetic risk profiles, and treatment disparities in the American Black PD population and use the results to develop guidance for educating Black patients and their treating clinicians on how to prevent racial disparities in PD and foster future research opportunities in racial disparities and improve management (Figure 1). This is a multicenter, cross-sectional, case-case, nonrandomized, observational study.

### Study Setting and Design

**Figure 2:**
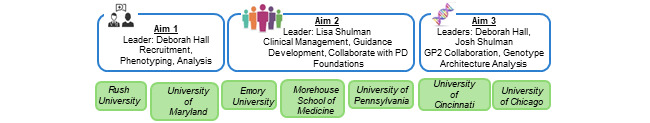
Overview of the organizational structure. GP2: Global Parkinson’s Genetic Program; PD: Parkinson disease.

The 7 study sites are Rush University, University of Maryland, University of Cincinnati, University of Pennsylvania, University of Chicago, Emory University, and Morehouse University (Figure 2). The sites were chosen based on US census data showing higher populations of Black persons in these geographic regions. A total of 400 Black patients with PD and 200 White patients with PD will be recruited. Patients who consent to enroll will have a single study visit encompassing data and sample collection for all 3 aims. A virtual option can also be used to complete questionnaires, followed by an in-person visit for the remaining measures.

### Eligibility Criteria

The eligibility criteria are intentionally broad to facilitate the inclusion of diverse samples and ensure the achievement of enrollment targets. Inclusion criteria include age >18 years and fulfilling the Movement Disorder Society Clinical Diagnostic Criteria for Parkinson’s Disease [20]. Patients must self-identify as being Black or White race. Patients must also be able to sign an evaluation to sign consent [21] or have a legally authorized representative (LAR) sign on their behalf. Exclusion criteria include insufficient English to complete study activities or inability to meet idiopathic PD criteria. Eligibility criteria are broad to include representative samples and ensure meeting enrollment targets.

### Recruitment and Screening Strategies

Recruitment will occur in the outpatient movement disorder clinics at the 7 study sites. Treatment clinicians or study coordinators will approach potential participants to discuss participation. Potential participants will be identified by prescreening medical records or by referral to the treating provider. Each White participant was initially matched to be within 5 years of age of the mean age of every 2 Black participants enrolled at the same site. Each time 2 Black participants were enrolled at a site, the study statistician would contact the site with the age range of the next White participant. Part-way through the study, propensity matching replaced this manual process. Potential participants will be interviewed to determine if they meet the eligibility criteria for enrollment.

Black patients with PD have been underrepresented in clinical research [22]. Therefore, study planning includes targeted strategies to facilitate recruitment and foster participant satisfaction with research participation. An advisory board of 6 individuals comprised of Black individuals with PD and family members of affected individuals assists in developing outreach strategies and providing feedback on their study visit experience, with protocol modifications as needed. The patient advisory board members will have participated in the study at 1 of the study sites. Educational strategies include initiatives for both study participants and study staff. A brief video and flip charts with culturally appropriate information are used to improve understanding of participation in research, including the informed consent process [23]. Participation of Black study coordinators is encouraged to foster rapport and cultural sensitivity. Teaching cross-cultural sensitivity is part of the prestudy and annual activities for all site investigators and coordinators [24]. Barriers to recruitment and enrollment will be explored, with each enrolled participant completing a Participations in Clinical Trials Questionnaire that includes a question on barriers to research participation [25]. During quarterly Steering Committee meetings with all site investigators, recent publications on minority recruitment and research are reviewed with the study author.

### Study Assessments and Outcome Measures

Study participants will have a single visit with the site investigator and coordinator after obtaining consent or a hybrid combination of in-person and virtual visits. Recommendations of the NINDS PD CDE Working Group informed the choice of measures, scales, and questionnaires. Data will be collected and entered into a REDCap (Research Electronic Data Capture) system that uses electronic data entry forms. General assessments include demographic data (age, sex, education, and income), medical history, and a confirmatory neurological examination. The remaining assessments fall into 3 categories, namely motor and nonmotor symptoms (Table 1), quality of life and social determinants of health (Table 2), and management of PD (Table 3, Multimedia Appendix 1).

**Table 1:** Motor and nonmotor measures for phenotyping.

Data type or domain and measure or instrument		Description
**Parkinson disease severity**		
	MDS-UPDRS^a^ parts I to IV^b^	Revised, improved version of the MDS-UPDRS for use in PD^c^ studies [26]
	Hoehn and Yahr stages	Common system for staging PD [27]
**Physical function**		
	2-minute walk, 4-meter gait speed, 9-hole pegboard, grip strength, and balance test^b^	To quantitate physical performance relative to available age and gender norms [28]
	Functional reach	A single-item assessment of balance [29]
	Schwab and England Activities of Daily Living Scale	A single-item rating of independent function [30]
	PROMIS Profile-29 Physical Function version 2.0 4a^b^	Measure of disability [31,32]
**Cognitive function**		
	Montreal Cognitive Assessment version 8.3^b^	Detects mild cognitive impairment in PD [33,34]
	Benton Judgment of Line Orientation 15-items	Measure of spatial perception and orientation [35,36]
	Hopkins Verbal Learning Test	Test of verbal short-term memory requiring rapid encoding of information [37]
	Digit span	Measure of verbal working memory [38]
	Semantic fluency	Measures semantic fluency using animal categories [39]
	Symbol Digit Modalities Test^b^	Screens for cognitive impairment [40]
**Mental health**		
	PROMIS Profile-29 depression, anxiety	Measures depression and anxiety
**Pain**		
	PROMIS Profile-29 Pain Interference version 1.1	Measures pain interference with daily life [41]
	PROMIS Profile-29 Pain Intensity version 1.0	Measures severity of pain
**Sleep**		
	Epworth Sleepiness Scale	Measures daytime sleepiness in adults [42]
	PROMIS Profile-29 version 1.0 Sleep Disturbance 4a, PROMIS Profile-29 version 1.0 Fatigue 4a	Measures sleep disturbance and fatigue [43,44]
**Autonomic**		
	Scales for Outcomes-PD Autonomic^b^	Measures autonomic function [45]

^a^MDS-UPDRS: Movement Disorder Society Unified Parkinson’s Disease Rating Scale.

^b^Minimum dataset.

^c^PD: Parkinson disease.

**Table 2:** Quality of life and patient health expectations measures.

Data type or domain and measure or instrument			Description
**Health-related quality of life**			
	The Parkinson’s Disease Questionnaire-8^a^	PD^b^-specific quality of life scale [46]	
**Patient health expectations**			
	Expectations Regarding Movement Scale	Expectations regarding movement with aging [6]	
**Participation in medical research**			
	Trust in Medical Researchers Scale	Likelihood of participation in medical research [47]	
**Social determinants**			
	PROMIS Informational Support version 2.0 4a	Measures access to information and resources [48]	
	Health Stressors Rush Survey	Covers food security, utilities, insurance, transportation, and housing instability	
	PROMIS Profile-29 Ability to Participate in Social Roles and Activities version 2.0 4a	Measures satisfaction with social roles and activities [48]	

^a^Minimum dataset.

^b^PD: Parkinson disease.

**Table 3:** Study assessments for management of Parkinson disease.

Data type or domain and measure or instrument		Description
**Pharmacological management**		
	Parkinson’s Disease Medications Questionnaire^a^	Antiparkinsonian drugs^a^
	Prescribed Non-Parkinson’s Medications Questionnaire^a^	Non-Parkinson drugs
	Unprescribed Drugs Questionnaire	Vitamins, dietary supplements, alternative therapies, recreational drugs, drugs of abuse
**Surgical management**		
	Surgical Questionnaire^a^	DBS^b^, FUS^c^
**Rehabilitation therapy**		
	Rehabilitation Referral Questionnaire^a^	Physical, occupational, speech therapy
	Godin Leisure-Time Exercise Questionnaire, Activity Questionnaire	Exercise and activity [49]
**Mental health and social services**		
	Mental Health and Social Services Referral Questionnaires^a^	Psychiatrist, psychologist, social worker
**Telehealth use and acceptance**		
	Telehealth Use and Acceptance Questionnaire^a^	Perceived usefulness and ease of use [50]
**Participation in clinical trials**		
	Participation in Clinical Trials Questionnaire	Clinical trial participation and barriers to participation
**History of treating clinicians for PD**		
	History of treating clinicians for Parkinson’s disease^a^	Number and types of clinicians, medical visit frequency
**Medical comorbidities**		
	Cumulative Illness Rating Scale-Geriatrics (CIRS-G)^a^	Medical comorbidities [51]
**Self-efficacy**		
	PROMIS Self-Efficacy for Management of Chronic Conditions	Self-efficacy for managing daily activities, symptoms, medications, and emotions [52]
**Health literacy**		
	Rapid Estimate of Adult Literacy in Medicine	Health literacy [53]
	eHealth Literacy Scale	eHealth literacy [54]

^a^Minimum dataset.

^b^DBS: deep brain stimulation.

^c^FUS: focused ultrasound.

Participants who used a LAR for consent can complete assessments with assistance from a research assistant or proxy (care partner). All scales and questionnaires include a check box to document proxy assistance. The scales are administered by the neurologist (site investigator), study coordinator, or other staff as appropriate. The investigator completes the following assessments: MDS-UPDRS [26], Hoehn and Yahr Staging [27], Schwab and England Activities of Daily Living Scale [55], and the Cumulative Illness Rating Scale [51]. The investigator will also complete a questionnaire for the GP2 study that includes demographic and disease-specific information, such as disease duration. The study coordinator administers the rest of the study assessments.

Participants have blood samples drawn and sent to the National Institutes of Health for genetic studies. All participants are also offered enrollment into the Parkinson’s Foundation PD GENEration study in which seven PD genes are examined and results are disclosed to the participant. If the participant consents, an additional blood sample is shipped to the PD GENEration sequencing vendor (Fulgent Genetics). For these participants, genetic counseling, including results disclosure, is performed by either a PD GENEration genetic counselor from Indiana University or the genetic counselor at the Rush University site (either live or through telemedicine).

To optimize recruitment, all Black patients with PD are eligible for enrollment regardless of physical or cognitive impairment level. Therefore, not all patients can complete all study assessments. A minimum dataset, including the primary outcome measures, must be completed, as well as the sample collected for all study participants. The recommended order of study assessments prioritizes outcome measures such that primary outcome measures are completed first, cognitive measures are completed early, and the minimum dataset for all study aims is collected. When a participant cannot complete all study assessments, the site investigator must be contacted. The investigator and coordinator should arrive at a consensus about what assessments will be omitted, and coordinators should record the omitted assessments. The patient can only complete selected study assessments (no proxy assistance permitted), including the cognitive assessments and the “Rapid Estimate of Adult Literacy in Medicine” [25].

### Outcome Measures

The primary outcome measures to characterize PD phenotypes include the NIH Toolbox Motor assessments (2-minute walk, 4-meter walk, balance test, pegboard test, and grip strength) [28]; the Montreal Cognitive Assessment (MoCA), Symbol Digit Modalities Test (SDMT), PROMIS Profile-29 Physical Function, Parkinson’s Disease Questionnaire-8 (PDQ-8), and the MDS-UPDRS. The primary outcome measures to assess PD clinical management include the proportion of participants with antiparkinsonian medications prescribed at the initial visit to the neurologist, the proportion of participants with initial treatment of depression, and the proportion of participants with PD surgical interventions. To characterize genetic risk variants in our sample, the frequencies of the 90 established common PD genetic risk variants based on published genome-wide association studies in a predominantly White population will initially be examined. The study team will also aggregate and summarize sequencing results from PD GENEration, including potential rare variants among 7 established PD genes. The proportion of LRRK2- and GBA-PD will be summarized based on either PD GENEration, which permits comprehensive detection of potential pathogenic alleles, or GP2 genotyping, which can detect many of the most common, recurrent pathogenic variants. Finally, exploratory genotype–phenotype analyses will determine whether established PD risk variants modify clinical manifestations in Black individuals.

### Sample Size

The sample size was calculated for each primary outcome measure described above with 80% power and a 2-sided test to detect a similar effect size observed from our pilot data (Table 4). Bonferroni correction was applied to control the overall significance level at .05. The minimal sample size for these analyses requires 365 Black and 183 White participants to complete this aim. The data observed in 3 studies guided the sample size calculation for PD clinical management (White vs non-White or Black: 78% vs 62% for measure 1 [4], 92% vs 80% for measure 2 [56], and 10% vs 0.4% for measure 3 [57]). The sample size was calculated using 90% power and a 2-sided test for each primary measure. Bonferroni correction was applied to control the overall significance level at .05. With a 1:2 ratio, the largest sample size required among all 3 measures is 173 White patients with PD and 346 Black patients with PD. For the genetic studies, our sample size will permit exploratory analyses, and all data will be contributed to GP2 for fully-powered meta-analysis.

**Table 4:** Pilot data effect sizes for sample size calculation.

Primary measures	White patients with PD^a^ (n=25), mean (SD)	Black patients with PD (n=25), mean (SD)
4-meter walk computed score	1.1 (0.2)	0.8 (0.3)
Balance *t* test score	47 (12)	32.7 (13.1)
Pegboard dominant score	33.2 (10.9)	41.4 (15.6)
MoCA^b^	27 (2)	23.3 (3.1)
SDMT^c^	49.6 (9.1)	39.4 (14.1)
PDQ-39^d^	12.8 (7.9)	22.5 (12.3)

^a^PD: Parkinson disease.

^b^MoCA: Montreal Cognitive Assessment.

^c^SDMT: Symbol Digit Modalities Test.

^d^PDQ-39: Parkinson’s Disease Questionnaire.

### Data Collection and Monitoring

The co-principal investigators (DH and LS), core administrative, and coordinating personnel meet weekly to identify potential issues with consent or assessment procedures, manage any reported adverse events, and monitor the sites’ progress. The REDCap database created to house the data is audited weekly to ensure fidelity. All study data are directly entered into the REDCap database in real time unless internet connectivity is an issue at the site.

### Statistical Analysis

Each primary outcome measure will be compared between Black and White patients with PD. Bonferroni correction will be applied. The primary outcome measures are a 4-meter walk computed score, Balance test *t* score, Pegboard dominant *t* score, MDS-UPDRS motor score [26], MoCA [33], SDMT [58], PROMIS Profile-29 Physical Function, and PDQ-8 [46]. For significant measures, linear regression analysis will be performed to examine further the difference between the 2 groups with adjustment for age, sex, and disease duration. The interaction effects of race with these 3 variables will be explored. Regression analysis for cognitive measures will adjust for depression and anxiety scales in addition to these demographics. Regression will also be adjusted for comorbidities using the Cumulative Illness Rating Scale-Geriatrics score. The study site effect will be controlled as a random effect in the model. All analyses will be done using SAS (version 9.4).

For the PD management analysis, each primary outcome measure will be compared between Black PD and White PD groups using the chi-square test or Fisher exact test. Bonferroni correction will be applied. A hierarchical logistic regression analysis will assess the determinants of racial disparities in management. Besides race, age, sex, and education, income, symptom severity, and comorbidities will be included in the model. The interaction effect of race with those variables will be explored. The study site effect will be controlled as a random effect in the model. Secondary analyses will investigate differences between White PD and Black PD groups in other clinical management measures, including telehealth, participation in clinical trials, and rehabilitation therapy.

### Genetic Analysis

This study will leverage a quality control and analysis pipeline established for the GP2 program. After standard quality control of raw genotyped data, data will be imputed to the most recent build of the multiethnic 1000 genomes reference panel using the default settings of miniMac2. This will yield ~15 million variants to test after additional quality control. These data will be contributed to GP2 for genome-wide association meta-analysis and admixture mapping. Within the RaDPD sample, ~90 currently established common PD risk variants to examine frequencies will be extracted. For analyses of PD genetic modifiers, linear regression—with an initial focus on outcome phenotypes with established evidence from the published literature, including age of onset, motor progression, and cognition—will be performed [59-65]. These outcomes will be supplemented with the most promising outcomes based on the results from aim 1 analyses identifying clinical features that differentiate PD in Black versus White patients. Sequencing results will be available for participants in PD GENEneration for 7 genes associated with PD: *GBA, LRRK2, PRKN, SNCA, PINK1,* *PARK7,* and *VPS35*. Comprehensive genome sequencing of all samples is planned for a future project.

### Ethical Considerations

This study was approved by the Rush Institutional Review Board (IRB; ORA 20121005), which serves as the single-site IRB for this study. Each participant will sign a consent form, which includes blood and genetic analysis, before participation. Each participant also signs permission for the DNA results from sequencing to be housed in the PDGeneration database and the National Institutes of Health (deidentified) through GP2. If the participant requests genetic results, they are identifiable to PDGeneration, facilitating the return of results. Potentially vulnerable study populations are patients with cognitive impairment and socioeconomic disadvantages. Given the study aims to investigate racial disparities in the phenotype–genotype differences between Black and White patients with PD, it is necessary to include these populations. Study participants must be able to provide informed consent as determined by the Evaluation to Sign Consent to confirm they understand the risks and benefits and can provide their informed consent. This low-risk study does not present a greater risk to potentially vulnerable populations. Given the short duration of this study (1 study visit), it is not expected that significant cognitive decline will occur throughout the study to impact a participant’s ability to provide ongoing consent. Participants unable to achieve a passing score on the Evaluation to Sign Consent will be excluded unless a LAR is present to provide consent.

A human subjects research ethics review will occur at the time of IRB approval. The informed consent process occurs with the study team live during the study visit. Data are entered directly into a password-protected, Health Insurance Portability and Accountability Act–compliant REDCaP database, with the participant having an assigned site-specific study ID. Participants are compensated US $100 for the study visit plus US $25 for travel expenses, and some sites arrange transportation with established institutional programs.

## Results

The Rush IRB approved the project as the single-site IRB in February 2022 and funded by NINDS in April 2022. Recruitment began in July 2022. At the time of submission of this manuscript, 131 participants had been enrolled. One of the original study sites has been replaced by a new site due to issues with regulatory approval. Other activities have included quarterly steering committee and monthly coordinator meetings. Steering committee meetings include discussing recent publications and speakers focused on racial disparities.

## Discussion

### Study Rationale

The study rationale is that the knowledge gained will improve clinical diagnosis and management for Black persons with PD, drive programming to improve access to care and management, and inform research strategies in PD in the Black community. It is anticipated that the main findings of this study will be that Black patients with PD will have more gait abnormalities and higher rates of genetic variants for PD specifically seen in the Black population. It is also anticipated that Black individuals with PD will be less likely to have been referred or have access to specialized care for PD, including surgical treatments and nonmedication therapies. This protocol was structured as a one-time visit to encourage participation and to optimize the likelihood of full data collection. Aim 3 is a collaborative effort with the Global Parkinson’s Genetics Program (GP2), a worldwide consortium funded by the Aligning Science Across Parkinson’s initiative to understand the genetic architecture of PD. This collaboration enables samples collected in this study to be part of a larger gene discovery effort with investigators from African and Afro-Caribbean patients with PD to foster a greater understanding of genetic variation specific to the Black population [17]. This study fills a unique niche by performing deep phenotyping to study racial disparities and analyze genotype–phenotype relationships in PD.

The recruitment of Black patients into research studies is historically much lower than White patients [22]. Contributory factors include distrust owing to historical research abuse and institutional racism, lack of information and understanding of research studies and informed consent, insufficient recruitment efforts by researchers, social stigma, and financial considerations [66]. In 1993, the National Institutes of Health established the Revitalization Act, which mandated minority inclusion in randomized clinical trials. An important strategy to promote study recruitment in both Black and White patients with PD is raising awareness of previous studies showing evidence of racial disparities in access to care, disease features, and clinical management.

A potential barrier to participation in PD research is motor and cognitive impairments, present in patients with PD of all racial and ethnic backgrounds. To accommodate the needs of patients with more severe motor and cognitive impairment, the protocol was designed to allow for the collection of a “minimum dataset.” The goal is to complete as many study assessments as possible. However, implementing more limited data collection will enable the successful completion of enrollment targets and ensure the inclusion of a representative range of disease severity. The typical time of the study visit is between 3 to 6 hours, and more limited data collection, focused on the primary outcome measures for each of the study aims, reduces the visit time to 1 to 2 hours.

### Conclusions

Many unique challenges arise in clinical research in the Black PD community, but the importance of understanding racial disparities warrants focus on this population. Given what little is known about how PD manifests in Black patients, this will be the most comprehensive study of the phenotype and management of PD in the US Black population, with the potential to improve clinical diagnosis and management and to foster future research directions. It is not clear what the true implications of the study will be, but improvement of study recruitment in this population, education of patients, caregivers, and clinicians on disparities, and ultimately, changes in practice will drive the final impact of the study and drive health policy recommendations at a national level.

## Appendix

Multimedia Appendix 1 Instruments, measures, and scale information.

Multimedia Appendix 2 Peer-review report from the Clinical Neuroscience and Neurodegeneration Study Section (CNN) - Brain Disorders and Clinical Neuroscience Integrated Review Group - Center for Scientific Review (National Institutes of Health, USA).

## Abbreviations

GP2: Global Parkinson’s Genetic Program
IRB: Institutional Review Board
LAR: legally authorized representative
MOCA: Montreal Cognitive Assessment
NINDS: National Institute of Neurological Disorders and Stroke
PD: Parkinson disease
PDQ-39: Parkinson’s Disease Quality of Life
REDCap: Research Electronic Data Capture
SDMT: Symbol Digit Modalities Test

## Data Availability

The datasets generated and analyzed during this study will be available upon reasonable request. These will be distributed to individual investigators on approval of the principal investigators (distribution costs covered by the requesting investigator). The study teams will use common data elements and a REDCap system to maximize the ability to share these data. Other products of the blood samples (serum, etc) may be shared if there are remaining samples. Research results will be disseminated publicly through publication in scientific journals and in the context of scientific meetings at which project findings will be reported. Such data publication will occur during the term of the project or after its conclusion, as appropriate and in keeping with normal scientific practices for reporting results.
