# Supplementary material for: Racial Disparities in Parkinson Disease Clinical Phenotype, Management, and Genetics: Protocol for a Prospective Observational Study
Source: JMIR Res Protoc. 2025 Apr 7;14:e60587. doi: 10.2196/60587 (PMC12012400; doi:10.2196/60587)
Supplement: Multimedia Appendix 1 [file resprot_v14i1e60587_app1.docx]

**Table 5.** Instruments, Measures and Scale Information.

| Clinical Outcome Assessment (COA) | COA Type | Time to Complete | Scoring System | Measurement Properties |
| --- | --- | --- | --- | --- |
| Movement Disorder Society Unified Parkinson’s Disease Rating Scale (MDS-UPDRS) | ClinRO | 30 minutes | 5-point Likert Scale (0-4)  Linear (no cut-offs) | Internal consistency: [26]  Part I: Adequate (α=0.79)  Part II: Excellent (α=0.90)  Part III: Excellent (α=0.93)  Part IV: Adequate (α =0.79)  Concurrent validity between UPDRS and MDS-UPDRS:  Part I: Excellent (*r*=0.76)  Part II: Excellent (*r*=0.92)  Part III: Excellent (*r*=0.96)  Part IV: Excellent (*r*=0.89) |
| Hoehn and Yahr Stages | ClinRO | 5 minutes | 6-point Likert Scale (0-5) | Studies document a moderate to significant level of inter-rater reliability, with nonweighted and weighted Kappa scores ranging between 0.44 and 0.71[67] |
| 2-min walk, 4-meter gait speed, 9-hole pegboard, grip strength, balance test | PerfO | 20 minutes | Linear (no cut-offs) | Overall good Test-retest reliability and criterion validity.[68] |
| Functional Reach | PerfO | 5 minutes | **Parkinson's Disease:**  Cut-off of 25.4 cm indicates fall risk (sensitivity of 30%), but a cut-off of 30.1 increases sensitivity to 56%  Cut-off of 25.4 cm indicates fall risk (specificity of 92%), and a cut-off of 30.1cm decreases specificity to 77%  <31.75 cm indicates fall risk (sensitivity of 0.86, specificity of 0.52 for risk of falling)[44] | Excellent test-retest reliability (ICC=0.84)[69]  Good predictive validity of Functional Reach Test at predicting maximum top, middle and bottom reaches for Parkinson’s Disease Patients (*r*=0.72, 0.76, and 0.73 respectively) [70] |
| Schwab & England Activities of Daily Living Scale | ObsRO | 5 minutes | Likert Scale: Rated in 10% increments:  1) 100% = completely independent  2) 0% = vegetative | Test/Retest Reliability: Adequate (ICC=0.7) [71]  Construct Validity: Moderate correlation with health related quality of life in PD patients.[72] |
| PROMIS Profile-29 Physical Function | PRO | 5-7 minutes | 0-30 (no cut-offs) | (several studies)  Excellent intenal consistency: α=>0.9 |
| Montreal Cognitive Assessment | PerfO | 10 minutes | 0-30. In PD the cut-off scores are:  Normal: ≥26  MCI: 22 -25  Dementia: ≤21 | High test-retest reliability: ICC=0.92, p<0.001  Excellent internal consistency (α=0.82)  Construct Validity:  high correlation with MMSE: r=0.87, p < 0.001[33] |
| Benton Judgment of Line Orientation 15-items | ClinRO | 5 minutes | 0-15 (no cut-offs) | Excellent intenal consistency: α=0.82 [50] |
| Hopkins Verbal Learning Test | ClinRO | 5 minutes | 0-12 (no cut-offs) | Poor performance on the test predicted cognitive decline.[73] |
| Digit Span (DS) | ClinRO | 1-3 minutes | Digit Span yields raw and scaled scores for total and subscores. Each section has a max raw score of 16; total max raw score is 48. (no cut-offs) | Split-half reliability α=0.93  Test-retest reliability ICC=0.83[74] |
| Semantic Fluency | ClinRO | 5 minutes | Linear (no cut-offs) | Poor performance on the test predicted cognitive decline.[73] |
| Symbol Digit Modalities Test | ClinRO | 5 minutes | 0-50 (no cut-offs) | Poor performance on the test predicted cognitive decline.[73] |
| PROMIS Profile-29 Depression, Anxiety | PRO | 5-7 minutes | 0-40 anxiety, 0-40 depression (no cut-offs) | Excellent intenal consistency: α=0.93  Good convergent validity with other PROMIS‐29 subscales[75] |
| PROMIS Profile-29 Pain Interference | PRO | 5-7 minutes | 0-40 (no cut-offs) | Excellent intenal consistency: α=0.96  Good convergent validity with other PROMIS‐29 subscales[75] |
| PROMIS Profile-29 Pain Intensity | PRO | 5-7 minutes | 0-15 (no cut-offs) | Excellent intenal consistency: α=>0.90  Good convergent validity with AS-legacy measures[76] |
| Epworth Sleepiness Scale | PRO | 5 minutes | 4-point Likert scale (0-3)  Score 0-24, cut-offs:  Normal range: 0-10  Excessive daytime sleepiness (EDS) >10  Mild EDS: 11-12  Moderate EDS: 13-15  Severe EDS: 16-24 | Intenal consistency varied between 0.73 and 0.90 (mean = 0.82) in ten separate investigations.  The ESS had a statistically significant association with self-rated problem sleepiness but low correlation with mean sleep latency (r=0.51) or measures of sleep apnea severity. |
| PROMIS Profile-29 Sleep Disturbance and Fatigue | PRO | 5-7 minutes | 0-40 (no cut-offs) | Good convergent validity with other PROMIS‐29 subscales[75] |
| Scales for Outcomes in PD Autonomic | PRO | 10-15 minutes | 5-ponit Likert scale (Never, sometimes, regularly, often). No cut-offs. | Test/Retest Reliability: Good (ICC=0.87)  Spearman correlation between the total score and HY stage was 0.60 (P<0.01)[59] |
| PDQ-8 | PRO | 5 minutes | 5-ponit Likert scale (0-4)  The summed score is divided by total possible score and given as a percentage score out of 100. (no cut-offs) | Excellent internal consistency: α=0.88[77]  Concurrent Validity PDQ-8 & PDQ-39: Excellent (r = 0.96)[35] |
| Expectations Regarding Movement Scale (EMR) | PRO | 5 minutes | 0-36 (no cut-offs) | Excellent internal consistency: α=0.90  Factor analysis supported construct validity of the ERM and HSB scores.[78] |
| Trust in Medical Researchers Scale (TIMS) | PRO | 7-8 minutes | 5-point Likert (0-48) No cut-offs | Excellent internal consistency: α=0.87  Race, education, health status, prior participation, and willingness to participate were each significantly associated with researcher trust.[79] |
| PROMIS Informational Support | PRO | 5-7 minutes | 0-40 (no cut-offs) | Excellent internal consistency: α=0.92  Worse social health is associate with low functioning.[80] |
| Health Stressors Rush Survey | PRO | 5 minutes | Qualitative (no cut-offs) | Not applicable |
| PROMIS Profile-29 Participation in Social roles | PRO | 5-7 minutes | 0-55 (no cut-offs) | Excellent internal consistency: α=0.95  Excellent concurrent validity between Ability to Participate in Social Roles & Activities Scale (Social Role Domain) of PROMIS-29 and Short Form-36 Social Domain (*r*=0.75) [81] |
| Parkinson’s Disease Medications Questionnaire | ClinRO | 10 minutes | Qualitative (no cut-offs) | Not applicable |
| Prescribed Non-Parkinson’s Medications Questionnaire | ClinRO | 10 minutes | Qualitative (no cut-offs) | Not applicable |
| Unprescribed Drugs Questionnaire | ClinRO | 5 minutes | Qualitative (no cut-offs) | Not applicable |
| Surgical Questionnaire | ClinRO | 3 minutes | Qualitative (no cut-offs) | Not applicable |
| Rehabilitation Referral Questionnaire | ClinRO | 3 minutes | Qualitative (no cut-offs) | Not applicable |
| Godin Leisure-Time Exercise Questionnaire (GLTEQ) | PRO | 5 minutes | Score ranges from 0 to 119 (>24 active, 14-23 moderately active, <14 sedentary | Excellent internal consistency: α=0.94  Two-week test-retest reliability coefficients were respectively 0.94, 0.46, 0.48, and 0.80 for self-reports of strenuous, moderate, light, and sweat-inducing exercise.[82] |
| Mental Health and Social Services Referral Questionnaires | ClinRO | 5 minutes | Qualitative (no cut-offs) | Not applicable |
| Telehealth Use and Acceptance Questionnaire | ClinRO | 3 minutes | Qualitative (no cut-offs) | Excellent internal consistency: α=0.94  Perceived ease of use had a positive relationship with future compliance (*P*=0.02) |
| Participation in Clinical Trials Questionnaire | ClinRO | 5 minutes | Qualitative (no cut-offs) | Not applicable |
| History of Treating Clinicians for Parkinson’s Disease | ClinRO | 3-4 minutes | Qualitative (no cut-offs) | Not applicable |
| Cumulative Illness Rating Scale- Geriatrics (CIRS-G) | ClinRO | 7-8 minutes | 0-56 (no cut-offs) | Interrater reliability between two physicians: ICC=0.81, 95% CI of 0.76–0.85[83]  Good divergent validity vis a vis functional disability in predicting mortality and hospitalization.[84] |
| PROMIS Self-Efficacy for Managing Chronic Conditions | PRO | 5-7 minutes | Linear (no cut-offs) | Significant correlations were seen between the Self-Efficacy measures and other PROMIS short forms (r>0.38)[64] |
| Rapid Estimate of Adult Literacy in Medicine (REALM) | ClinRO | 5 minutes | 0-7, <6 at risk for poor health literacy | Concurrent validity between REALM and 7 REALM-SF was r=0.96[85] |
| eHealth Literacy Scale (eHEALS) | ClinRO | 7 minutes | 0-50 (no cut-offs) | Excellent internal consistency: α=0.88  eHealth literacy levels were not related to overall self-evaluations of health and were not a significant predictor of perceived health status over time in this sample.[66] |

Key: α: Chronbach’s alpha | ClinRO: Clinician-Reported Outcome Measure | PRO: Patient-Reported Outcome Measure | ObsRO: Observer-Reported Outcome Measure | PerfO: Performance Outcome Measure | MCI: Mild Cognitive Impairement | MMSE: Mini-Mental Status Examination | HSB: Healthcare Seeking Beliefs for parkinsonism | CI: confidence interval | AS: Ankylosing Spondylitis
