# Supplementary material for: Racial Disparities in Parkinson Disease Clinical Phenotype, Management, and Genetics: Protocol for a Prospective Observational Study
Source: JMIR Res Protoc. 2025 Apr 7;14:e60587. doi: 10.2196/60587 (PMC12012400; doi:10.2196/60587)
Supplement: Multimedia Appendix 2 [file resprot_v14i1e60587_app2.pdf]

1R01NS125294-01 HALL, DEBORAH

RESUME AND SUMMARY OF DISCUSSION: This is a very highly significant and timely application that aims to study Parkinson's disease (PD) in Blacks, testing the premise that PD has different clinical phenotypes in Blacks versus Whites, due to a combination of suboptimal clinical management and intrinsic genetic modifiers. The application raised a great deal of enthusiasm from two of the reviewers. There is some evidence that the incidence of PD in Blacks is much lower than that in Whites, but it is unclear if this observation is due to health inequities or has a biological foundation. Moreover, pilot studies from the group suggest that the disease is more severe, which leads to greater impact on quality of life, and limited therapeutic options. Thus, there is a pressing need for studies like this, to inform research strategies, improve diagnosis and access to care, and to better drive clinical management of PD in Blacks. The application comes from an outstanding strong group of experienced investigators from seven different institutions, with successful history of collaboration, with sites carefully selected to reach Black communities. One reviewer however reasoned that recruitment could be further facilitated by the addition of investigators from a strong historically black medical school. Along this line, another reviewer also commented that power calculations and recruitment expectations may be optimistic. The proposal is well written, with extensive, robust preliminary studies, supporting the aims. Methods and statistical analyses are well described, and pitfalls discussed. However better consideration for the possibility of other risk factors, unrelated to race, driving the outcomes would strengthen the analyses. One of the reviewers was less enthusiastic, raising concern that the cross-sectional design has limited ability to inform the causes of racial differences in outcomes; moreover, some of the proposed analyses are confirmatory, providing limited new information. There were also some question on the analytical plan, where some of test seemed not ideal, but, overall, the statistical issues were considered addressable. After discussion, the reviewers reached a consensus that, even if some weaknesses may be appreciated in the approach, the impact of the application will be high, due to the public health significance and the potential to help bridging gaps in care and knowledge of PD in Blacks.

DESCRIPTION (provided by applicant): Parkinson disease (PD) is a progressive, disabling neurological disorder. Studies investigating the features of the disease in Black populations are uncommon, with some suggesting that Blacks with PD are more disabled, with greater disease severity, and with different clinical features compared to White PD patients. These health disparities are likely to influence the quality of care for AA with PD. The specific aims of this study are to investigate 1) PD symptoms and signs in Black participants, 2) the management of PD in these participants, and 3) to determine genotype-phenotype relationships. Based on prior studies, the main study hypotheses are that Blacks with PD have more severe motor features, greater impact on their quality of life, less therapeutic options, and dissimilar genetic variation compared to Whites with PD. Aim 1 investigates motor, non-motor, and quality of life scales in 400 Black and 200 White participants recruited at seven different US sites. Aim 2 investigates management in the same participants including medication, non-medication and surgical treatments. As part of this Aim, clinical guidelines will be developed for PD treating clinicians to raise awareness of racial disparities in PD. In collaboration with PD foundations, educational programming will be developed for the Black PD community to improve self-management skills and reduce disparities. In Aim 3, a collaboration with the Global Parkinson's Genetics Program will be utilized to determine genotype-phenotype relationships in the Black participants. The overall goal of this study is to investigate racial disparities in Blacks with PD by studying key components of the disease and then disseminate the findings to the neurologic and patient community with targeted education and guidance. This study will result in new scientific knowledge with in-depth characterization of racial disparities in PD and will change clinical practice by raising awareness of differences in the PD clinical phenotype, patient-reported outcomes, and PD recognition and management. The results will

also impact public health since recognition of differences in disease manifestations and management in Black patients will improve healthcare delivery and quality of care in this underserved and understudied population.

PUBLIC HEALTH RELEVANCE: Parkinson disease (PD) is a progressive, disabling neurological disorder, but the impact of the disease on Black patients with PD is unclear due to less research in this population. This study investigates racial disparities by studying symptoms and signs, management, and genotype relationships in Black PD participants in comparison to a group of White PD participants. This study will result in new scientific knowledge regarding PD in the Black population by characterizing the basis for racial disparities, raising awareness of differences in PD recognition and management, and educating patients in this underserved and understudied population.

CRITIQUE 1

Significance: 3
Investigator(s): 3
Innovation: 3
Approach: 3
Environment: 2

Overall Impact:

There is little known about Parkinson's disease (PD) in the black population. The incidence is thought to be half of whites. The reasons are not known. Pilot data suggests that in Blacks who have PD- they may be more severe. The proposed work is built on a long history at Rush in this area. The team has the skills to do the work and represents investigators with a strong history of success in PD studies. The proposal is strong as aim 1 and 2 will sort out issues related to health equity as it related to the assertion that Black patients have more severe disease. Getting accurate data about the onset, progression, and treatment of Black patient with PD will have on going impact for years. The additional genetic data could also answer important questions. Environmental data will need to be collected in aims 1 and 2 to judge the influence. This is an outstanding and committed team and impactful work that will provide data for many future studies.

1. Significance:

Strengths

- There is an unmet need to understand PD in Black patients and how best to assess, treat and overcome barriers for those with PD.
- There is a need to understand if the lower incidence rate is artificial due to health inequity issues or is part of a genetic background.
- The project will address important health goals, education and develop recruitment paradigms for expanding black patients in PD trials.

Weaknesses

- A small percentage of PD is known to be genetic. It is not clear how the genetic and clinical drivers of worse PD in Black will be sorted out. However, Aim 1 and 2 are powerful stand alone as important information- even if the genetic data of risk factors comparing black and non-black patients with PD does not materialize.

2. Investigator(s):

Strengths

- Dr. Hall has deep experience in phenotyping and has assembled a team from 7 institutions to contribute. All are experienced investigators.
- There is a history of the team working together successful in the past.
- Dr. Barnes contribution on the project is significant as to collect data on health equity and outcomes
- Well described structure.

Weaknesses

- An addition from a one of the strong historically black medical schools would have added diversity to the team and enhanced the proposal. Many have been successful in being trusted messengers and could improve recruitment. While the schools in the group of 7 are powerful in recruitment, adding an HBCU would send a strong message.

3. Innovation:

Strengths

- Asks an important and bold question about PD in Black patients. Results will be a natural history, case based assessment that could impact many future intervention trials.

Weaknesses

- The tools are standard in the PD work and all seem to be in person.

4. Approach:

Strengths

- An advisory team representing Black patients with PD is important.
- Addressed historical barriers to recruitment
- Two pilot studies with one with a large N using quantitative assessments
- Qualitative comments from the pilot studies are powerful.
- Tables clearly outline the assessment plan which encompasses motor, non- motor, and quality of life
- The work for aim 1 and 2 are well described and supported by the pilot data and prior published work.
- Aim 3 partners with other existing GP2 funding.
- Detailed statistical plan for each aim.
- Pitfalls of each aim are addressed.

Weaknesses

- Aim 3 should also analyze the data without race. Minor weakness the results of aim 3 may be known or new risks for PD that are separate from race. Race may have no role in the genetics

of PD. Aim 2 and 3 may find that health inequities are the main drivers of the difference in outcomes.

5. Environment:

Strengths

- All of the sites are very strong in movement disorders and have facilities to see the patients.

Weaknesses

Protections for Human Subjects:

Acceptable Risks and/or Adequate Protections

Data and Safety Monitoring Plan (Applicable for Clinical Trials Only):

Not Applicable (No Clinical Trials)

Inclusion Plans:

- Sex/Gender: Distribution justified scientifically
- Race/Ethnicity: Distribution justified scientifically
- For NIH-Defined Phase III trials, Plans for valid design and analysis:
- Inclusion/Exclusion Based on Age: Distribution justified scientifically

Vertebrate Animals:

Not Applicable (No Vertebrate Animals)

Biohazards:

Not Applicable (No Biohazards)

Applications from Foreign Organizations:

Not Applicable (No Foreign Organizations)

Resource Sharing Plans:

Not Applicable (No Relevant Resources)

Multiple PD/PI Leadership Plan:

Acceptable

Authentication of Key Biological and/or Chemical Resources:

Not Applicable (No Relevant Resources)

Budget and Period of Support:

Recommend as Requested

CRITIQUE 2

Significance: 1
Investigator(s): 1
Innovation: 1
Approach: 2
Environment: 1

Overall Impact:

The goals of this MPI R01 from a group of outstanding investigators is to profile phenotypic differences in PD Black people compared to PD White people (Aim 1) and determine potential causes of these differences, including disparities in clinical management (Aim 2) and/or from a distinct spectrum of genetic risk variants (Aim 3). The main hypothesis is that phenotypic differences in PD are due to a combination of suboptimal clinical management and intrinsic genetic modifiers in Black versus White populations The goal is that knowledge gained from this project will improve clinical diagnosis and management for Blacks with PD, drive programming to improve access to care and management, and inform research strategies in PD. This is a well written proposal from an outstanding team, with extensive careful preliminary studies and rigorous experimental and statistical methods. I believe that the results of this study will help to advance public health gaps in care for this group of people and will advance our knowledge in the way PD affects the Black community.

1. Significance:

Strengths

- Investigating underlying phenotypic differences between the Black and White population in carefully controlled studies will have a significant impact
- Understanding whether genetic variants discovered in White PD populations are also present and influential in Black PD populations is an important scientific unmet need
- Choosing sites centered in communities where Black people get healthcare and working to improve access, communication and therapy is a strength for the success of the proposal and for healthcare delivery in general
- Preliminary data: high impact of developing a community advisory group who will guide and contribute to the project

Weaknesses

- None

2. Investigator(s):

Strengths

- outstanding investigators and strategically placed
- clear credibility for each Aim

Weaknesses

- None

3. Innovation:

Strengths

- The comprehensive focus on Black PD people and encompassing the community as well as a diverse healthcare team to address the areas of unmet need is innovative

Weaknesses

- None

4. Approach:

Strengths

- Comprehensive Aims and assessments across several sites was well written
- Excellent preliminary data allowed detailed power analyses and credible sample sizes

Weaknesses

- None

5. Environment:

Strengths

- Outstanding

Weaknesses

- None

Protections for Human Subjects:

Acceptable Risks and/or Adequate Protections

Data and Safety Monitoring Plan (Applicable for Clinical Trials Only):

Not Applicable (No Clinical Trials)

Inclusion Plans:

- Sex/Gender: Distribution justified scientifically
- Race/Ethnicity: Distribution justified scientifically
- For NIH-Defined Phase III trials, Plans for valid design and analysis:
- Inclusion/Exclusion Based on Age: Distribution justified scientifically

Vertebrate Animals:

Not Applicable (No Vertebrate Animals)

Biohazards:

Not Applicable (No Biohazards)

Applications from Foreign Organizations:

Not Applicable (No Foreign Organizations)

Resource Sharing Plans:

Acceptable

Authentication of Key Biological and/or Chemical Resources:

Not Applicable (No Relevant Resources)

Budget and Period of Support:

Recommend as Requested

CRITIQUE 3

Significance: 2
Investigator(s): 1
Innovation: 3
Approach: 4
Environment: 1

Overall Impact:

While the prevalence of Parkinson's disease (PD) is lower in African-Americans compared to Whites, African-Americans tend to exhibit more severe disease and greater degrees of disability. Explanations for these differences are currently lacking, largely because African-Americans have been underrepresented in PD research, especially so for genetic studies. This proposal aims to address this gap and fully characterize the phenotypic, clinical management, and genetic differences between African-Americans and Whites with PD. The investigators propose to recruit 400 African-Americans and 200 Whites with PD from 7 centers in the US. Participants with then be phenotyped across a range of domains including motor function, cognition, and disability, with genetic samples being contributed to a large PD consortium. Strengths of the application include a very strong investigative team with significant collaborative experience, a focus on health equity for African-Americans with PD, and leveraging resources from the Global Parkinson's Genetics Program for genotyping and larger-scale consortium genetic analyses. Weaknesses of the proposal stem from its cross-sectional nature, implying that analyses will be largely descriptive with limited to ability to "explain" racial differences in outcomes, in line with what REGARDS has done in stroke. I wondered whether a more valuable study would have been to focus entirely on African-Americans with longitudinal follow-up to look at progression and other changes over time, recognizing that the present proposal would certainly setup such a longitudinal continuation. For example, I fully expect that AAs with PD with exhibit lower cognitive (MoCA) scores as compared to Whites. This is a known feature of the MoCA when used in diverse populations, and so it's unclear how this will be informative for PD specifically, unless there is some examination of change in cognitive function over time? Second, it was not clear how Specific Aim

2 will add to the background data provided concerning the clinical management of PD in African-Americans. There seems to be decent existing data, some from the investigative team, showing that African-Americans have reduced access to care including telemedicine, and tend to be treated less aggressively. So what will Aim 2 add other than confirming these disparities? Third, recruitment will span the bulk of the grant. While this is likely appropriately realistic, it doesn't leave room for recruitment lags, and the contingency plans were generally fairly vague. Finally, the power calculations and statistical analysis plan are ok, but have issues. The power calculations are probably optimistic, being based on small pilot data and not factoring in the complexity of the actual proposed multivariable analytic strategy. The analytic plan has a number of (mostly addressable) issues: Aim 1 describes using linear regression and yet includes a site random effect? Many of the proposed scales for Aim 1 can often be heavily skewed with floor/ceiling effects, and this isn't addressed. GCTA doesn't actually estimate heritability (it estimates the percent of genetic variance tagged by common variation).

Protections for Human Subjects:

Acceptable Risks and/or Adequate Protections

Data and Safety Monitoring Plan (Applicable for Clinical Trials Only):

Not Applicable (No Clinical Trials)

Inclusion Plans:

- Sex/Gender: Distribution justified scientifically
- Race/Ethnicity: Distribution justified scientifically
- For NIH-Defined Phase III trials, Plans for valid design and analysis: Not applicable
- Inclusion/Exclusion Based on Age: Distribution justified scientifically

Vertebrate Animals:

Not Applicable (No Vertebrate Animals)

Biohazards:

Not Applicable (No Biohazards)

Applications from Foreign Organizations:

Not Applicable (No Foreign Organizations)

Resource Sharing Plans:

Acceptable

- Would have liked to see more detail about where data will specifically be deposited publicly

Authentication of Key Biological and/or Chemical Resources:

Not Applicable (No Relevant Resources)

Budget and Period of Support:

Recommend as Requested

THE FOLLOWING SECTIONS WERE PREPARED BY THE SCIENTIFIC REVIEW OFFICER TO SUMMARIZE THE OUTCOME OF DISCUSSIONS OF THE REVIEW COMMITTEE, OR REVIEWERS' WRITTEN CRITIQUES, ON THE FOLLOWING ISSUES:

PROTECTION OF HUMAN SUBJECTS: ACCEPTABLE

INCLUSION OF WOMEN PLAN: ACCEPTABLE-BOTH GENDERS

INCLUSION OF MINORITIES PLAN: ACCEPTABLE-MINORITIES AND NON MINORITIES

INCLUSION ACROSS THE LIFESPAN: ACCEPTABLE-NO CHILDREN INCLUDED

COMMITTEE BUDGET RECOMMENDATIONS: The budget was recommended as requested.

Footnotes for 1 R01 NS125294-01; PI Name: HALL, DEBORAH A

NIH has modified its policy regarding the receipt of resubmissions (amended applications).See Guide Notice NOT-OD-18-197 at https://grants.nih.gov/grants/guide/notice-files/NOT-OD-18-197.html. The impact/priority score is calculated after discussion of an application by averaging the overall scores (1-9) given by all voting reviewers on the committee and multiplying by 10. The criterion scores are submitted prior to the meeting by the individual reviewers assigned to an application, and are not discussed specifically at the review meeting or calculated into the overall impact score. Some applications also receive a percentile ranking. For details on the review process, see http://grants.nih.gov/grants/peer_review_process.htm#scoring.
